# Extracellular Vesicles in Feto–Maternal Crosstalk and Pregnancy Disorders

**DOI:** 10.3390/ijms21062120

**Published:** 2020-03-19

**Authors:** Danilo Buca, Giuseppina Bologna, Alice D’Amico, Sara Cugini, Francesca Musca, Melania Febbo, Dolores D’Arcangelo, Davide Buca, Pasquale Simeone, Marco Liberati, Ester Vitacolonna, Sebastiano Miscia, Francesco D’Antonio, Paola Lanuti

**Affiliations:** 1 Department of Obstetrics and Gynecology, University of Chieti, 66100 Chieti, Italy; alicedamico91@gmail.com (A.D.); liberati@unich.it (M.L.); 2 Department of Medicine and Aging Sciences, University “G. d’Annunzio” of Chieti-Pescara, 66100 Chieti, Italy; giuseppina.bologna@hotmail.it (G.B.); simeone.pasquale@gmail.com (P.S.); evitacolonna@unich.it (E.V.); s.miscia@unich.it (S.M.); p.lanuti@unich.it (P.L.); 3 Centre on Aging Sciences and Translational Medicine (Ce.S.I.-Me.T.), University “G. D’Annunzio” of Chieti-Pescara, 66100 Chieti, Italy; 4 School of Medicine and Health Science, G. d’Annunzio University of Chieti-Pescara, 66100 Chieti, Italy; saracugini@hotmail.it (S.C.); franc3scamusca@gmail.com (F.M.); melania.febbo@gmail.com (M.F.); dolores.darcangelo@gmail.com (D.D.); 5 Department of Molecular Medicine and Development, University of Siena, 53100 Siena, Italy; bucadavide@gmail.com; 6 Fetal Medicine Unit, Department of Medical and Surgical Sciences, Department of Obstetrics and Gynecology, University of Foggia, 71121 Foggia, Italy; dantoniofra@gmail.com

**Keywords:** placental extracellular vesicles, syncytiotrophoblast, preterm-labor, pre-eclampsia, gestational diabetes mellitus, fetal growth restriction

## Abstract

Extracellular vesicles (EVs) actively participate in inter-cellular crosstalk and have progressively emerged as key players of organized communities of cells within multicellular organisms in health and disease. For these reasons, EVs are attracting the attention of many investigators across different biomedical fields. In this scenario, the possibility to study specific placental-derived EVs in the maternal peripheral blood may open novel perspectives in the development of new early biomarkers for major obstetric pathological conditions. Here we reviewed the involvement of EVs in feto–maternal crosstalk mechanisms, both in physiological and pathological conditions (preeclampsia, fetal growth restriction, preterm labor, gestational diabetes mellitus), also underlining the usefulness of EV characterization in maternal–fetal medicine.

## 1. Introduction

Extracellular vesicles (EVs) are membrane-bound organelles, released into the extracellular milieu by all cell types (i.e., endothelial cells, platelets, and leukocytes). EVs also carry specific cargoes consisting of lipids, proteins, RNAs, micro-RNAs, and DNA fragments, and larger EVs may also contain whole organelles, such as mitochondria. It also has been demonstrated that EVs retain a vast enzymatic repertoire and mediate a wide range of biological activities, even after the release from their parental cells [1]. EVs largely take part of the normal physiological cell-to-cell communication process and recent studies have shown that different EV subtypes can display peculiar regulatory functions, mediated both by their surface receptors and their content [2]. For these reasons, EVs are emerging as relevant players in inter-cellular crosstalk, in a multitude of pathophysiological conditions, including bacteria–host interaction, cancer progression, cardiovascular, neurological, proteinopathies-driven neurodegenerative disorders, metabolic, autoimmune diseases, as well as fetal–maternal communication [1,3,4,5,6,7,8,9,10].

It is interesting to note that EVs are implicated in embryo implantation, placentation, pregnancy maintenance, and pregnancy complications. The potential utility of EVs in early diagnosis, prevention, and prognosis of some pregnancy pathologies, such as preeclampsia, abortion, intrauterine growth restriction (IUGR), and gestational diabetes, has been largely underlined and here reviewed.

## 2. Extracellular Vesicles Subtypes

In the past decade, different EV subtypes have been identified and classified on the basis of their different structures, biochemical properties, and biogenesis mechanisms. EVs are able to convey specific cargoes to target cells at different distances from the site where they originate. It has been demonstrated that the EV cargo is specifically packaged and depends on the features of the parental cells, as well as on the microenvironment in which they are produced, or on the stimuli that had induced their release. In any case, the EV cargo may include different combinations of lipid components, genetic material (DNA fragments, RNAs, and miRNAs), proteins, and even small organelles, such as mitochondria, that were found to be conveyed by larger EVs [1,11,12]. 

It has been observed that EVs can be released during physiological events in vivo, such as cell growth, proliferation, activation, apoptosis, or senescence. Furthermore, some conditions, such as oxidative or shear stress, inflammation, senescence, and cell death, may also induce EV production [13,14,15,16,17,18]. In any case, EVs are released into the extracellular milieu and finally they could reach the blood circulation. EVs were identified in many different body fluids, such as cerebrospinal fluid, tears, saliva, urine, milk, and peripheral blood [1,6,8,19,20,21,22,23]. For all of these reasons, EVs were pointed out as a reliable source of biomarkers.

On the other hand, it has been demonstrated that EVs may have strong effects on their target cells [2,24]. Therefore, EVs have been described as key players of inter-cellular crosstalk and have been implicated in a variety of mechanisms, such as signal transduction, metastasis development, and even fetal–maternal interactions [3,4,5,7,25,26,27,28]. 

**Figure 1:**
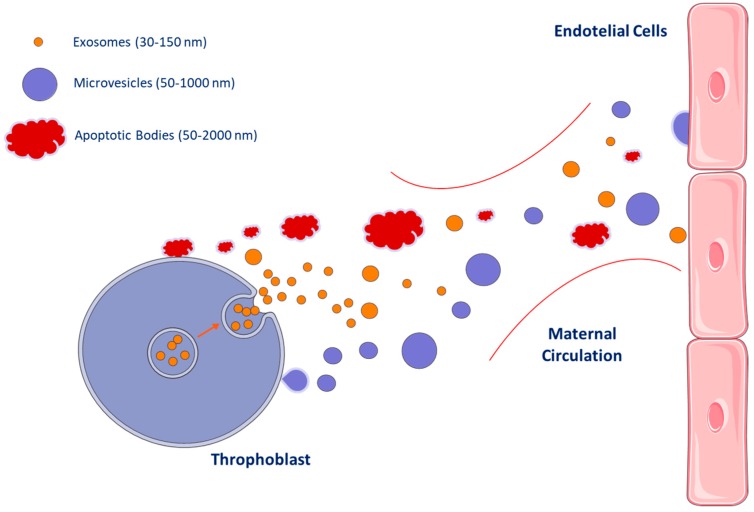
Extracellular vesicle sub-types released from placenta in normal pregnancies. Three main subsets of extracellular vesicles (EVs), with overlapping dimensions, have been traditionally described: Exosomes (orange, 30–150 nm) stem from the intracellular endosomal compartments and express tetraspanins; microvesicles (blue, 50–1000 nm) are produced by budding and express the phenotype of their parental cells; and apoptotic bodies (red, 50–2000 nm) are released by cells undergoing apoptosis and express phospatydilserine on their surface. As shown, the three EV subsets display overlapping dimensions. Under normal conditions, small numbers of EVs are released from the placenta and reach the maternal circulation. EVs participate in the crosstalk between feto–placental and mother tissues, with a relevant exchange of information. The appropriate function of those EVs guarantees successful pregnancies, as well as the healthy fetal development. This figure has been created from Servier Medical Art, licensed under Creative Common Attribution 3.0 Generic License http://smart.servier.com/.

Three main subtypes of EVs were traditionally described: exosomes, microvesicles (MVs), and apoptotic bodies [29,30] (Figure 1).

Exosomes are small vesicles (approximately 50–100 nm in diameter) produced from multivesicular bodies. Exosomes are gathering to play a crucial role in different cell–cell interaction processes (i.e., antigen presentation, signal transduction, and immune response), in view of their ability to carry specific complex cargo of proteins, nucleic acids, and lipids [28].

Microvesicles, also known as microparticles or ectosomes, are vesicles of 50–1000 nm in diameter [29] surrounded by a double phospholipid layer [31]. They are released by budding/blabbing of the plasma membrane and express the phenotype of their parental cells. Several sub-types of microvesicles deriving from leukocytes, platelets, red blood cells, neurons, and mesenchymal stem cells, as well as from cancer stem cells have been identified and described (Figure 1) [4,22,30]. It has been observed that, when microvesicles are released by activated cells, the phosphatidylserine is flopped to the outer leaflet of the plasma membrane bilayer. For this reason, it exists as a subset of microvesicles that can be identified for the positivity to Annexin V, which is a molecule able to bind phosphatidylserine [4,22,32]. Phosphatidylserine is also expressed by apoptotic body membranes, and for this reason, the positivity to Annexin V (which is related to the surface exposure of phosphatidylserine) is not a useful method that allows to distinguish apoptotic bodies and microvesicles derived from activated cells. Apoptotic bodies must be identified, instead, for their positivity to the caspase substrates [33]. 

More recently, a specific tumor-derived microvesicle subtype, larger than normal microvesicles and known as large oncosomes [5], has been described. It has been demonstrated that large oncosomes are involved in the process leading to cancer cell migration and metastasis [30].

Apoptotic bodies are membrane vesicles of approximately 50–2000 nm in diameter, released during the programmed cell apoptosis processes (Figure 1) [30,34]. Given that apoptotic cells express phosphatidylserine on their surface [35], the positivity to Annexin V (which binds phosphatidylserine) has been considered a hallmark of apoptotic bodies, even if the enrichment in caspase 3 and 7 has been proposed to be more specific for apoptotic bodies [33]. It has been recently demonstrated that, as with all the other EV subtypes, apoptotic bodies represent an active vehicle of intercellular communication from dying to living cells [14].

This EV classification has been largely used in the past, but it generated confusion among investigators, since a plethora of terms, based on different criteria, have been used to identify the different subtypes of EVs. Furthermore, it has been recently observed that EV subpopulations display overlapping dimensions. For these reasons, in order to avoid confusion, the International Society of Extracellular Vesicles (ISEV) established, in a recent position paper, that EVs should be classified as small EVs if they are smaller than 100–200 nm in diameter and medium/large if their diameters are larger than 200 nm [36]. It has been demonstrated that EVs are released after different stimuli, and given that they are constantly present in body fluids (i.e., peripheral blood), circulating EVs have been proposed as a suitable source of liquid biopsy, able to provide reliable information, being indices of cell activation and/or tissue degeneration, and occurring during pathophysiological events in vivo [5].

## 3. Methods to Study and Measure EVs

Different methods have been applied for EV studies, as also summarized by the recent ISEV position paper [36,37,38]. It must be specified that, due to their small size, protocols for EV detection are based on EV enrichment procedures, such as centrifugation/ultracentrifugation, size exclusion chromatography, ultrafiltration, immunocapture, hydrostatic, or hydrostatic filtration dialysis. For all of these reasons, the EV’s final characterization is usually done on samples that differ, in terms of EV composition, from that of the original body fluid [37,39]. For these reasons, no consensus and standardization on the EV analysis has been reached. Actually, polychromatic flow cytometry (PFC) has been identified as a highly promising technique for EV characterization and enumeration, given that it analyzes thousands of events in a short period of time and multiple parameters simultaneously and it is also able to characterize and quantify EVs stemming from different parental cells [38]. EVs are in fact characterized by the phenotype of the parental cells, and, for example, given that endothelial cells express the marker CD31, also known as platelet endothelial cell adhesion molecule (PECAM1) [40,41]; therefore, endothelial-derived EVs are identified by the surface expression of CD31 [36]. In the same way, because mesenchymal stem cells expose CD90 [42,43,44], it has been observed that their derived vesicles are recognized by the positivity to CD90, which is a glycophosphatidylinositol cell surface protein, originally discovered as a thymocyte antigen [36].

However, flow cytometers are not sensitive enough to detect small EVs. More recently, it has been shown that smaller EVs can be detected by applying a simplified flow cytometry analysis able to directly detect EVs from fresh body fluids [20]. Combining such a detection methodology with the use of a tracer for the staining of the whole EV circulating population may allow the placement of the trigger threshold on a fluorescent channel, and therefore the identification of the smallest EVs become possible [45]. It also has been purposed to stain EV samples with phalloidin, in order to exclude damaged EVs from the flow cytometry analysis, allowing a more accurate discrimination of intact vesicles [6,19,20,46]. Finally, the use of the Rosetta Calibration system, which permits the identification of the EV compartment on the basis of the particle size values, combined to a flow cytometry standardized procedure, significantly enhance flow cytometry sensitivity [47,48]. 

All these new findings will open new perspectives in EV flow cytometry studies. Interestingly, concerning the gynecological filed, placenta-derived EVs are detectable in the maternal circulation, indicating that EVs may represent an intriguing target of future studies on pregnancy disorders [20]. In particular, it is well known that the EV cargo, consisting of genomic DNA fragments, RNAs, mRNAs, miRNAs, proteins, and lipids, reflect the characteristics of the cells of origin. For these reasons, the analysis and characterization of circulating EV phenotypes and cargoes could be seminal to provide a real-time picture of feto–maternal interactions. The intrapartum period represents the time during pregnancy when the feto–maternal relationship is challenged to the highest degree. Therefore, during this period, the monitoring of the maternal peripheral blood concentration and cargoes of placental-derived EVs by the recently developed flow cytometry methods has a high potential as dynamic measurable and accessible source of biomarkers. 

## 4. Extracellular Vesicles as Biomarkers

The EVs are constantly detected in a number of body fluids, as mentioned above [20]. In this scenario, the possibility to identify, characterize, and isolate specific placental-derived EVs in the maternal peripheral blood may open novel perspectives on the development of new early biomarkers for the major obstetric pathological conditions, including preterm-labor, pre-eclampsia, gestational diabetes mellitus, and fetal growth restriction.

Feto–maternal communication human placental syncytiotrophoblast (STB) is an epithelium that covers the entire surface of human placental villi, responsible for maternal–fetal exchange, and separating maternal and fetal blood compartments. STB forms the outer surface of the placental villous tree. Its apical plasma membrane faces the maternal intervillous space and its basal plasma membrane the endothelium of the fetal capillaries. The feto–maternal immunological interface is composed of maternal decidual cells that develop from the endometrium and the fetal trophoblast cells. In detail, three distinct sub-populations of decidual cells have been described: the cytotrophoblasts, the multi-nucleated syncytiotrophoblast (STBs), and the invasive extra-villous trophoblasts (EVTs) [49]. It represents the largest maternal–fetal interface, responsible for nutrient uptake, gas exchange, waste removal, protein and steroid hormone production, modulation of maternal physiology, and immune tolerance. The STB is unique in being human leukocyte antigen (HLA) null and therefore immunologically inert to prevent allorecognition and rejection by maternal T cells [49].

The STB plays a primary role in feto–maternal communication mechanisms, and it was demonstrated that the major source of placenta-derived EVs is the STB itself [50]. It is known that the placenta releases EVs into the maternal bloodstream, early in pregnancy, and small EVs were detected for six weeks of gestation [51]. Circulating EVs interact with their specific target cell via receptor binding, fusion, or various internalization routes, being able to sustain the activation of a number of signaling pathways that influence the recipient cell functions. It was demonstrated that EVs may be active mediators that communicate between the maternal endometrium and the embryo during implantation, deputy to regulating endometrial remodeling. EVs may also induce the release of proinflammatory cytokines so as to suppress the activation of natural killer cells and macrophages, altering inflammatory responses during pregnancy. MHC class I-related molecules downregulated Natural killer cell receptor functional Fas ligand and TRAIL molecules, the HLA-G and B7 family of immunomodulators isolated from first trimester placental tissues [49,52]. Trophoblast-derived EVs, passing through the uterine vein, reach the maternal circulation following the trophoblast deportation mechanism. Given that the trophoblastic surface antigens are not detected in retroplacental cord blood, trophoblast deportation is possibly restricted to the maternal aspect of the human placenta. However, it must be underlined that many studies have shown the presence of EVs in amniotic fluid samples [53,54,55]. 

The STB communicates with the maternal immune system using both soluble factors, such as chemokines, cytokines, and steroid and protein hormones, as well as other factors carried by EVs.

As a matter of fact, different markers were described for the detection of placental-derived EVs. Among them, STB-derived EVs expose the placental alkaline phosphatase (PLAP), which is co-expressed with endothelial nitric oxide synthase (eNOS3) in both small and medium/large STB-EV compartments. Conversely, small EVs resulted enriched in some surface molecules, such as the major histocompatibility complex class II (MHC class II), some members of the tetraspanin family, such as CD9, CD37, CD53, CD63, CD81, and CD82, and they use to express the endosomal sorting complex proteins, Apoptosis-linked gene 2-interacting protein X (Alix), Tumor susceptibility gene 101 protein (TSG101), and chaperones, while resulting negatives for the 94 kDa glucose-regulated protein (Grp94) [56]. In particular, the tetraspanins CD9 and CD63 play relevant roles in exosome genesis; therefore, they are usually used as exosome markers. CD37 is involved in T-cell–B-cell interactions, while CD53 and CD81 play a role in the regulation of cell development, activation, growth and motility, and in B-cell physiology, respectively. CD82 has been shown to be downregulated in tumor progression of human cancers. Small EVs are also enriched in glycoproteins and transmembrane proteins, such as integrins, glycoprotein Ib (GPIb), and P-selectin. Syntenin has also been described as an EV marker in this context [57]. On the other hand, several “eat me” proteins that represent molecules for phagocytes to identify and engulf dying cells or EVs, such as Annexin V and calreticulin, expressed by all the STB-EV fractions, and a number of “don’t eat me” markers, such as CD47 and CD31, have been demonstrated to play a pivotal role during pregnancy [58].

Interestingly, miR-210, a hypoxia-induced miRNA, recruiting Hypoxia Inducible Factor 1 Subunit Alpha (HIF-1α) and participating to angiogenesis and cell migration processes, is carried by EVs derived from extravillous and villous trophoblasts. It has been demonstrated that miR-210 is one of the most highly expressed miRNAs in the placenta as well as in the maternal blood [52,59,60]. Syncytiotrophoblast-derived EVs, staining positive for PLAP, were analyzed for several tRNA species in normal pregnancies. In this setting, a differential enrichment of individual tRNA species was observed in medium-large and small EVs. Most of the found tRNAs resulted in 5′-tRNA halves and it was demonstrated that 5′-tRNA halves interfere with the protein synthesis, suggesting that they may play a role in feto–maternal signaling. It is also been shown that NEP (also known as CD10 or neprilysin)-positive/PLAP-positive small EVs circulate in the placental blood [49].

It is noteworthy to mention that trophoblastic cells, which are rich in Epidermal Growth Factor Receptor (EGFR), release PLAP+/EGFR+ EVs in the maternal circulation during the first trimester of pregnancy. Placenta-derived small EVs, staining positive for EGFR, should be considered as a possible cause of endothelial dysfunction in women with preeclampsia [61].

All of these data indicate that there is a plethora of recognized markers for placental derived EV identification and analysis, but consensus on a standardized EV phenotype for further clinical studies on placental EV-based biomarkers still represent an urgent need.

## 5. Extracellular Vesicle Roles During Pregnancy

EVs have been also implicated, during pregnancy, in a number of activities that we have reviewed here below.

Pregnancy is a state in which the maternal immune system transiently tolerates the antigens of the developing fetus minimizing the risk of rejection/abortion, meanwhile protecting the body against external pathogens. EVs play significant roles in modulating the maternal immunity for successful pregnancy. This represents a complex scenario of immunomodulation aimed at maintaining the efficiency in pathogens elimination without harming the fetus [49,62].

The secretion of small EVs is a potential mechanism by which placenta evades the cytotoxic effect of the maternal immune system and modulates the immune tolerance to the fetal antigens. Fas ligand (FasL) and TNF-related apoptosis-inducing ligand (TRAIL) are secreted in the active form by the STB-derived small EVs and mediate apoptosis of T cells offering immunotolerance to fetus [63]. Moreover, STB-EVs increased the T-dependent production of interferon γ [63,64] and transcription 3 phosphorylation in T cells in vitro [65]. Additionally, STB-EVs downregulate the T cell proliferation induced by phytohemagglutinin [64]. Monocytes participate to this process by binding and internalizing STB-derived EVs [63,65,66], and finally producing some cytokines, such as tumor necrosis factor α (TNF-α) and interleukin (IL)-1-β [63,65,66]. Furthermore, ligands activating NKG2D receptors on NK cells and cytotoxic T cells are expressed and secreted in small EVs. Some B7 immune-regulatory ligands, which bind to receptors in lymphocytes are released in the small EVs [49]. Even neutrophils seem to be activated by STB-EVs in vitro [67].

It has been demonstrated that the transmission of some viruses (CMV, HSV, and coxsackie B3) to the endothelial and the fibroblast compartments is mediated through the miRNA of the chromosome 19 miRNA cluster (C19MC), which induces autophagy in recipient cells, limiting the infection. C19MC is an imprinted primate-specific miRNA cluster expressed exclusively in the human placenta [68]; in particular, miR-512-3p and miR-517-3p are carried by placenta-derived small EVs. Another miRNA, miR-141 carried by STB-derived small EVs, suppressed T-cell proliferation and it is involved in regulation of activation of the nitric oxide/cGMP signaling pathway. Activated eNOS, the main isoform of nitric oxide transported by STB-EVs, is expressed in all types of STB-derived EVs. During normal pregnancy, the number/weight of small EVs is greater than medium/large EVs. Small EVs are considered anti-inflammatory, while medium/large EVs are proinflammatory [69]. 

In contrast to the immune-suppressive role of small EVs, some reports illustrate the pro-inflammatory functions of small EVs that are able to increase the recruitment of monocytes from the maternal system into the fetal–maternal interface and increase the release of pro-inflammatory cytokines [49]. 

The three complement regulatory proteins, membrane cofactor protein (MCP; CD46), decay-accelerating factor (DAF; CD55), and protectin (CD59), are expressed by the trophoblastic-derived EVs. Interestingly, DAF is a phosphatidylinositol (PI)-anchored protein, localized to the brush border of the human syncytiotrophoblast [70]. It was shown that both DAF and MCP regulate T cell functions independently from their complement regulatory roles. Therefore, these proteins carried by trophoblastic EVs may produce a dual function, being able to prevent complement activation, while EVs are free-floating in the maternal circulation, and regulating the function of the maternal T cells that might otherwise produce adverse immune responses against paternally derived antigens expressed by the placenta [58]. 

These data show that EVs play a significant role in modulating the maternal immunity and permit immune tolerance to the fetal antigens minimizing the risk of rejection/abortion. EVs play also an important role in limiting the viral infection (CMV, HSV, and coxsackie B3) that can usually lead to serious fetal pathologies. 

## 6. Potential Usefulness of Extracellular Vesicles in Maternal–Fetal Medicine

EV patterns resulted in modifications in a number of obstetric clinical conditions. For these reasons, EVs have a high potential as dynamic, noninvasive maternal biomarkers. As reviewed here below, changes in EV subtypes, as well as some correlations with clinical indices, have been reported in preeclampsia and growth restriction (Table 1). 

### 6.1. Preeclampsia (PE)

A number of studies have demonstrated that placental-secreted EVs have different implications in the pathogenesis of preeclampsia (PE) [52,60]. It is known that toxic/dangerous factors deriving from the placenta, including EVs, act as triggers for PE by activating the cells of the maternal endothelium before the symptom onset. As a matter of fact, the levels of STB-EVs in maternal plasma are increased in PE compared to normal pregnancy, where they play pro-inflammatory, anti-angiogenic, and pro-coagulant functions. Placenta samples of patients with preeclampsia display lower levels of STB-EV, carrying eNOS and reduced NO activity compared to control placentas; this may contribute to the reduction of synthesis and bioavailability of NO in this pathology. An increased release of placental STB-EVs and free fetal hemoglobin (HbF) into the maternal circulation have been demonstrated in PE. This condition may lead to endothelial cell re-programming, causing detrimental cellular functions, such as arterial stiffness [78]. This phenomenon may explain the long-term cardiovascular consequences in women who have suffered of PE during pregnancy [72]. It has been demonstrated that EVs from placental explants treated with preeclamptic sera significantly increase intercellular adhesion molecule 1 (ICAM-1) and high mobility group box 1 (HMGB1) expression on endothelial cells [79]. It is well known that PE is associated with a significant platelet activation. Kohli et al. showed that EVs induce thrombo-inflammatory responses specifically in the placenta. Activated maternal platelets cause NLRP3-inflammasome activation in trophoblast cells [71]. The inflammasome activation in trophoblast cells triggers a PE-like phenotype, characterized by pregnancy failure, elevated blood pressure, increased plasma soluble fms-like tyrosine kinase 1 (sFlt-1), and renal dysfunction. Inflammasome activation in trophoblast cells of women affected by preeclampsia corroborates the translational significance of these findings. The level of circulating total-miRNAs and hsa-miR-210 carried by EVs was increased in women with PE compared to those in healthy pregnancies. In addition, it was shown that hsa-miR-210 is secreted via small EVs, which may have a role in the pathophysiological mechanisms of the disease [75]. It has been also shown that miR-126 was decreased in both umbilical endothelial progenitor cells and in placentas from preeclamptic pregnancies, where miR-126 down regulates the expression of the anti-angiogenic gene Phosphoinositide-3-Kinase Regulatory Subunit 2 (PIK3R2) that is involved in the negative regulation of PI3K-Akt signaling pathways. It was demonstrated that STB-EVs from explant cultures of preeclamptic placentae induced increased secretion of proinflammatory cytokines, such as IL1-β, IL-6, IL-17, macrophage inhibitory protein-1-α and -β, or TNF-α in peripheral blood mononuclear cells compared to STB-EVs from normal placenta explants [80]. Additionally, STB-EVs from preeclamptic placental explants increased the response of peripheral blood mononuclear cells to lipopolysaccharide, while STB-EVs from normal placental explants suppressed the response of peripheral blood mononuclear cells to lipopolysaccharide [80].

This information may suggest that STB-EVs from preeclamptic placentae may be involved in the exaggerated inflammatory response that characterizes PE patients. STB-EVs play pro-inflammatory, anti-angiogenic, and pro-coagulant functions, which involve reduced NO activity, endothelial cell re-programming and arterial stiffness, platelet activation, inflammasome activation, and increased blood mononuclear cells to lipopolysaccharide.

### 6.2. Gestational Diabetes Mellitus (GDM)

Gestational diabetes mellitus (GDM) is defined as diabetes diagnosed in the second or third trimester of pregnancy that was not clearly overt diabetes prior to gestation [81]. It is known that GDM rapidly resolves after the delivery, confirming the active role of the placenta in its pathophysiology. The involvement of placental hormones (PH) released from the human placenta has been associated with the development of insulin resistance and gestational diabetes mellitus (GDM). In such a context, higher levels of circulating small EVs and STBEV-EVs were detected in GDM patients when they were compared to healthy women [76,82]. It was also observed that the contribution of placental small EVs expressing PLAP as a placental marker was comparatively lower in GDM pregnancies. Therefore, the ratio of small EV PLAP+/total small EVs was lower in GDM compared to normal pregnancies [55]. Interestingly, it has been reported that, in normal pregnancies, during the third trimester, the concentration of placental-derived small EVs is positively correlated with placental weight [83], and in GDM, placental mass increases compared to normal pregnancies. Interestingly, the concentration of total small EVs resulted negatively correlated with placental weight at the at late gestation deliveries (32–33 weeks). It is, therefore, possible to hypothesize a role for those placental-derived EVs for the identification of asymptomatic women who will develop GDM, that, in this way, could be diagnosed and treated earlier, between 11–14 weeks of gestation [55]. In GDM patients, EVs are characterized by specific cargoes. Interestingly, normal human placenta releases STB-EVs that carry biologically active dipeptidyl peptidase IV (DPPIV), which plays a role in type 2 diabetes by breaking down glucagon-like peptide 1 (GLP-1), which in turn regulates glucose-dependent insulin secretion. Furthermore, STB-EVs from GDM perfused placentae showed greater dipeptidyl peptidase-4 (DPPIV) activity, and DPPIV-bound STB-EVs increase eightfold in the circulation of women with GDM [76]. It has been demonstrated that amnion epithelial cells (AECs) produce small EVs that interact with myometrial, decidual, and placental cells (BeWo). Moreover, the treatment with AEC-derived small EVs increases the production of pro-labor inflammatory molecules (i.e., IL-6, IL-8, and Prostaglandin E2), inducing the aviation of NF-κβ in maternal myometrial and decidual cells [73]. Some other proteins were differentially carried by EVs from GDM patients and healthy women, such as the proteins involved in metabolic processes and in the biological regulations. Among them, spectrin alpha erythrocytic (SPTA)-1, CAMK2β, PAPP-A, Perilipin 4, fatty acid binding protein (FABP) 4, and hexokinase-3 were particularly abundant in EVs from GDM patients. Interestingly, pappalisin-1 (PAPP-A) and protein kinase II beta calcium/calmodulin-dependent (CAMK2β), two proteins involved in the regulation of insulin sensitivity, were downregulated and upregulated, respectively, in the small EVs isolated from GDM pregnancies. Interestingly, a negative correlation (*p* = 0.0135) between the maternal BMI and the concentration of EV-carried PAPP-A was demonstrated [82]. Different data showed modified EV features in GDM in terms of carried miRNAs. In detail, an up-regulation of miR-326 was demonstrated in EVs from GDM patients when compared to healthy women. miR-36 was negatively correlated to the levels of its target, the adiponectin. Some other miRNAs (miR-122-5p; miR-132-3p; miR-1323; miR‒136-5p; miR-182-3p; miR-210-3p; miR-29a-3p; miR-29b-3p; miR-342-3p; and miR-520h) were significantly upregulated in GDM patients than in healthy controls. In this context, it has been also shown that, during pregnancy, the abovementioned miRNAs are involved in proliferation, trophoblast differentiation, insulin secretion and regulation, and glucose transport mechanisms [77]. It has been also demonstrated that the hypertrophic adipose tissue of GDM patients produce differential expression of small EV-carried miRNAs, contributing to the systemic inflammatory state, to insulin resistance (IR), and also impacting on the placental metabolism, nutrient uptake, and deregulation of placental nutrient signaling pathways observed in obese GDM patients [84].

GDM pathogenesis is a multifactorial condition in which placental hormones play a relevant role. Therefore, some studies have focused on assessing the role of placental EVs in GDM, demonstrating their role in insulin secretion mechanisms, as well as in producing the systemic inflammatory state.

### 6.3. Other Complications

#### 6.3.1. Preterm Labor (PTL)

Preterm birth (PTB) is childbirth occurring at <37 completed weeks of gestation. Preterm delivery is still one of the biggest problems in obstetrics and has remained the major contributor to neonatal morbidity and mortality globally, accounting for 70% of neonatal deaths. In most cases, it occurs unexpectedly in women at low risk; therefore, the identification of a biomarker able to predict spontaneous preterm delivery poses a significant challenge due to the diversity of clinical presentations. Preterm delivery is the consequence of four main mechanisms: activation of the maternal–fetal placental interaction with the hypothalamic–pituitary–adrenal axis, inflammation in the amniochorionic-decidual tissue, decidual hemorrhage, and pathological distention of the myometrium [85].

It has been shown, by some authors, that EV dysregulation may have an impact also in the case of preterm labor (PTL). In detail, in EVs, the chromosome 14 miRNA cluster (C14MC) and the chromosome 19 miRNA cluster (C19MC) were generally decreased in PTL patients compared to normal gestations [74].

#### 6.3.2. Receptivity Failure

For a successful pregnancy, the synchronic coordination between the embryonic development and the endometrial status is crucial. The endometrium is a hormonally regulated organ. Endometrial receptivity refers to a hormone-limited period in which the endometrial tissue acquires a functional status allowing blastocyst implantation and therefore pregnancy initiation. It has been shown that, from the first trimester, small EVs, acting through their miRNAs, modulate communications between embryonic cells of the blastocyst and endometrial cells, promoting the receptivity of one and the adhesion of the other. In detail, it has been demonstrated that miRNA210 could be used as an index of a good implant, therefore possibly predicting an eventual receptivity failure [86].

## 7. Conclusions

The EV-mediated inter-cellular crosstalk, specifically produced by EV cargoes (i.e., genomic DNA fragments, RNAs, mRNAs, miRNAs, proteins, and lipids), is implicated in embryo implantation, placentation, pregnancy maintenance and, even, in pregnancy complications [49]. On the other hand, growing evidence show that placental EVs interact with a multitude of maternal immune and endothelial cells and influence their function [69]. In such a context, the potential utility of studying the EV maternal concentrations and cargoes in early diagnosis, prevention, and prognosis of some pregnancy pathologies, such as preeclampsia, abortion, intrauterine growth restriction (IUGR), and gestational diabetes, has been largely underlined [51].

Altogether these data show that the relevance of the EVs and, in particular, that of the STB-derived EVs in establishing and maintaining a physiological pregnancy, as well as in participating in the patho-physiological mechanisms of pregnancy pathology, is not fully understood yet. Further studies may offer novel perspectives to investigated on the mechanism, role, and potential of biomarkers of EVs during pregnancy.

## Figures and Tables

**Table 1:** General characteristics of the studies included in the review.

Author	Year	Study Design	Markers Described	Subtypes of EVs Described	Role	Implication	Potential Usefulness
Tong [54]	2015	Review	C19MC miRNA	STB exosomes	Inducing autophagy	Resistance to infection (coxsackie B3, herpes simplex, cytomegalovirus)	NS
Escudero [52]	2016	Review	miR-126, miR-17, miR-18, miR-19, miR-92, and miR-210	EVs	endothelial dysfunction	Preeclampsia	NS
Tannetta [57]	2016	NS	PLAP	STB-EVs	Placental marker	Preeclampsia	maintenance of healthy pregnancy
Tong [58]	2016	NS	CD47	macro-, micro- and nano- vesicles	determining if maternal cells can internalize trophoblastic vesicles	Preeclampsia	NS
			CD31	macro-, micro- and nano- vesicles	determining if maternal cells can internalize trophoblastic vesicles	Preeclampsia	NS
Kohli [71]	2016	NS	sFlt-1	EVs	Antiangiogenic properties. Platelet activation. Procoagulant function. Inflammasome activation.	Preeclampsia. Renal dysfunctions and proteinuria. Hypertension. Endothelial dysfunction. IUGR. Immune system dysregulation. Placental sterile inflammation. Pregnancy failure.	Early diagnosis, predictive and prognostic value.
Salomon [55]	2017	NS	CD63	exosomes	metabolic state of placenta	GDM	potentiality to develop a non-invasive biopsy of the placenta for early diagnosis and clinical management
Göhner [60]	2017	NS	NS	Placental STB-derived small EVs	Exaggerated inflammatory state	Preeclampsia	NS
Motta-Mejia [69]	2017	retrospective study	eNOS	all types of STBEV	Vascular status. Medium/large EVs are proinflammatory	Preeclampsia	Potential link between abnormal placental function and altered maternal vascular status
Cronqvist [72]	2017	Retrospective study	NS	Placental STB-derived EVs	Endothelial cell re-programming. Arterial stiffness	Preeclampsia	NS
Nair [49]	2018	Review	TSG101	Exosomes	EVs biogenesis and secretion.		
			ALIX	Exosomes	EVs biogenesis and secretion.		
			MHC II	MVs	Immune tolerance of mother to fetal allograft. Downregulation of NK cell cytotoxic functions.	Rejection/Abortion	
Hadley [73]	2018	NS	/	AEC small EVs	production of pro-labor inflammatory molecules (i.e., IL-6, IL-8 and PGE2)	Preterm Labor	NS
Fallen [74]	2018	NS	C14MC miRNA	EVs	decreased in PTL patients	Preterm Labor	NS
			C19MC miRNA	EVs	decreased in PTL patients	Preterm Labor	NS
Kaminski [56]	2019	Review	CD9	Exosomes	EVs biogenesis. Cell signaling mediate embryo growth and promote embryo implantation. Immune regulation.	Rejection/Abortion. Fetal death. Preterm birth. IUGR	
			CD63	Exosomes	EVs biogenesis. Cell Signaling: control trophoblast physiology which can promote embryo implantation. Immune regulation.	Rejection/Abortion	
			CD81	Exosomes	EVs biogenesis. Modulation of immune response. Feto–maternal tolerance. Embryo growth.	Rejection/Abortion. IUGR	
Gill [59]	2019	Retrospective study	NEP+/PLAP+	STB-derived small EVs	Inactivation of bioactive peptides	Preeclampsia	NS
Clemente [61]	2019	Retrospective study	EGFR+/PLAP+	Small EVs	Endothelial dysfunction	Preeclampsia	NS
Birò [75]	2019	Retrospective study	hsa-miR-210	Exosomes	Disturbed trophoblast invasion	Preeclampsia	NS
Kandzija [76]	2019	cohort study	DPPIV	STB-EVs	insulin-resistance	GDM	potentiality to regulate maternal insulin secretion
Gillet [77]	2019	NS	miR-326; miR-122-5p; miR-132-3p; miR-1323; miR-136-5p; miR-182-3p; miR-210-3p; miR-29a-3p; miR-29b-3p; miR-342-3p and miR-520	Small EVs	Proliferation. Trophoblast differentiation. Insulin secretion and regulation. Glucose transport mechanisms.	GDM	NS

In this table are collected data concerning the vesicle markers described in the various studies, roles of the EVs in the pathogenesis of the most important obstetric pathologies, and the potential use in clinical practice of EVs as biomarkers. C19MCmiRNa (chromosome 19miRNA cluster), CD (cluster of differentiation), PLAP (placental alkaline phosphatase), sFlt-1 (soluble fms-like tyrosine kinase 1), eNOS (endothelial nitric oxide synthase), TSG 101 (tumor susceptibility gene 101), ALIX (apoptosis-linked gene 2 interacting protein X), MHC II (major histocompatibility complex class II), NEP (neprilysin), EGFR (epidermal growth factor receptor), DPPIV (dipeptidyl peptidase IV), STB (syncytiotrophoblast), EV (extracellular vesicles), MV (microvesicles), AEC (amnion epithelial cells).
